# EP04 Is this scleroderma lung or COVID-19 lung?

**DOI:** 10.1093/rap/rkaa052.003

**Published:** 2020-11-03

**Authors:** Dr Saadia Malik, Dr Hadi Younas, Dr Loay Al Dhahir

**Affiliations:** Dr Sulaiman Al Habib Medical Group, Riyadh, Saudi Arabia

## Abstract

**Case report - Introduction:**

This is a case of Pakistani female with limited systemic sclerosis and associated mild interstitial lung disease. The lung disease was complicated by SARS-COV-2 related pneumonitis in April 2020 and that led to treatment challenges.

She was previously seen in multiple private hospitals and labelled as Rheumatoid arthritis. She was being treated with long term steroids and Methotrexate. After her initial presentation to our Rheumatology services, her diagnosis was correctly revised to Systemic Sclerosis with phenotype of CREST. Her treatment was adjusted to Vasodilators and Mycophenolate due to skin and Lung involvement.

**Case report - Case description:**

This is a case of 40-year-old Pakistani female who had been having multiple joint pains since 2010. She also experienced severe Raynaud’s.

She presented to our Rheumatology clinic in December 2018. Her symptoms included recurrent digital ulcers, tight and tough skin at fingers and Raynaud’s worse during winter months. Her examination confirmed peripheral cyanosis with multiple digital ulcers with superimposed infection, marked sclerodactyly and calcinosis. She was started on Vasodilator therapy including calcium channel blocker and PDE5 inhibitor due to severity of ulceration. Infection was managed with prolonged course of antimicrobial therapy. Her immunology showed positive anti nRNP/Sm. Anti-centromere and anti Scl 70 were negative. Her condition fit description of CREST (Calcinosis, RP, Oesophageal dysmotility, telangiectasia). Her management included weaning off Methotrexate and reduction in the dose of corticosteroids.

In February 2019, Respiratory work up showed normal Chest radiograph, High resolution CT chest showing no significant abnormality and FEV1 82%, FVC 86%, and DLCO 77%. Her PASP was 25mmHg. Overall, her condition remained stable over the course of next year. Her medication included Cellcept, low dose prednisolone, hydroxychloroquine, and Sildenafil. More importantly, Digital ulcers have been well controlled with combined vasodilator therapy.

In April 2020, she developed SARS-CoV-2 with mild respiratory symptoms and was admitted to a different hospital. Fortunately, she responded well to ward based supportive and symptomatic treatment with no need for respiratory support. Subsequently, she has seen a different respiratory physician and had repeat imaging of chest which has led to dilemma whether the ground glass opacities in both lungs is due to scleroderma lung or COVID-19 related lung disease. She was given high dose prednisolone by the respiratory physician which has been reduced in rheumatology clinic. The new findings on chest imaging are sequelae of SARS-COV-2.

**Case report - Discussion:**

This case highlights few important points as below:

1. Systemic sclerosis diagnosis was not made for many years even though she has had severe digital ulcers for a long time. She was being managed as Rheumatoid arthritis. Systemic sclerosis remains a difficult disease to diagnose and is still under recognised.
2. SARS-COV-2 related illness has not affected this patient adversely despite the fact of being on long term maintenance prednisolone of 7.5mg daily dose and Cellcept 2gm. Her cellcept was temporarily stopped during acute illness.
3. We know that viral pneumonitis can present with typical ground glass opacities in bilateral areas of lungs and differential diagnosis does include connective tissue related lung disease but this lady had no significant respiratory involvement prior to COVID-19 illness and follow up scan will help to decide if this is disease progression or related to viral cause.

**Case report - Key learning points:**

There are multiple learning points in this case:

1. Continuity of care under same primary team can avoid confusion related to diagnosis and diagnosis related complications. This lady had none, or mild subclinical lung involvement related to systemic sclerosis prior to contracting COVID-19 illness. Her CT chest findings after the episode of SARS-COV-2 were attributed to systemic sclerosis as she was seen by different respiratory team. This continuity is not always possible, but MDT collaboration needs to be improved across hospitals and across various departments.
2. Systemic sclerosis remains an under diagnosed and under recognized complex rheumatic disorder and more primary care physicians need to be educated so they can appropriately refer these cases to Rheumatology services.
3. Multi-disciplinary collaboration between Rheumatology, Respiratory and other specialties is the key point to manage these complex cases.
4. This case also highlights an interesting observation that presence of significant immune disorder and immunosuppressant medication does not always equate to worse outcome if patient contracts SARS-COV-2. Supportive care, appropriate observation, and temporary suspension of DMARD in such cases can avoid any further complications.

